# First person – Sofia de Oliveira

**DOI:** 10.1242/dmm.045401

**Published:** 2020-05-11

## Abstract

First Person is a series of interviews with the first authors of a selection of papers published in Disease Models & Mechanisms, helping early-career researchers promote themselves alongside their papers. Sofia de Oliveira is first author on ‘DnaJ-PKAc fusion induces liver inflammation in a zebrafish model of fibrolamellar carcinoma’, published in DMM. Sofia conducted the research described in this article while a postdoctoral fellow in Anna Huttenlocher's lab at the University of Wisconsin-Madison, Madison, WI, USA. She is now an assistant professor at Albert Einstein College of Medicine, Bronx, NY, USA, investigating the impact of Western-type diets on inflammation, and how such effect modulates the progression of different human diseases, particularly liver diseases and infectious diseases.


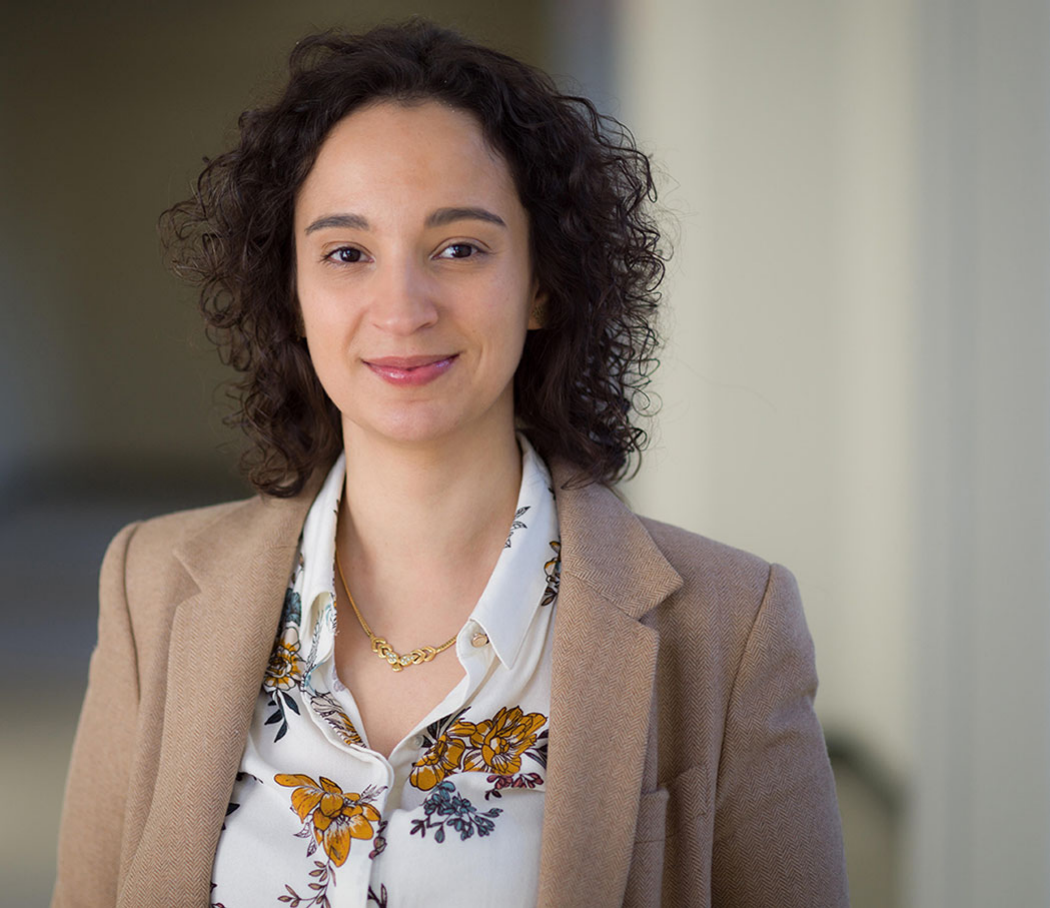


**Sofia de Oliveira**

**How would you explain the main findings of your paper to non-scientific family and friends?**

Fibrolamellar carcinoma (FLC) is a rare type of liver cancer that affects children and young adults. There are just two mouse models that allow *in vivo* studies of this disease. However, such models do not allow the *in vivo* study of the tumor microenvironment through non-invasive live imaging. In this study, we developed an FLC zebrafish model that allows the *in vivo* visualization of the immune cells in the liver microenvironment by non-invasive live imaging. Using this model, we found that inflammation is involved in the progression of this cancer at early stages. In addition, we found that Caspase-a (zebrafish homolog of caspase-1) is active in the liver tissue of zebrafish larvae with FLC, suggesting that inflammasome activation might be playing a role in FLC progression. Importantly, our study also found that pharmaceutical inhibition of TNFα and caspase-1 might be valuable targets for the development of future therapeutic approaches to treat FLC patients.

**What are the potential implications of these results for your field of research?**

There is currently no cure for FLC. Liver resection or transplant are the only options for FLC patients; recurrence rate is extremely high and the therapeutic options available are scarce and not efficient. To develop better therapeutic options for FLC patients we need to understand the biology of FLC. Immunotherapeutic approaches are extremely appealing nowadays; however, to make good use of such tools or develop new ones we need to understand how DnaJ-PKAc (the chimeric transcript *DNAJB1-PRKACA*) affects immune cell infiltration and polarization in the liver. Our work has provided a zebrafish FLC model that allows such studies. We have also provided the field with immune cellular (neutrophils and macrophages) and molecular (TNFα and caspase-1) targets that might be playing a role in FLC progression and that might be easily targeted by pharmacological approaches.

**What are the main advantages and drawbacks of the model system you have used as it relates to the disease you are investigating?**

We are using, for the first time, a small vertebrate animal model – the zebrafish – to study FLC biology. For us, the main advantage of our system is its transparency, which allowed us to visualize and investigate the interaction between immune cells and transformed hepatocytes *in vivo* by non-invasive imaging. The high fecundity rate of the zebrafish also makes it a perfect platform to quickly and efficiently perform drug screenings and find potential drugs to treat FLC in the near future. The major drawback was the low tumor incidence we had at adult stages that might be associated with zebrafish whole-genome duplication, or the need for a stronger promoter to overexpress the fusion transcript.

**What has surprised you the most while conducting your research?**

The level of liver inflammation we found at early stages of FLC. In theory, DnaJ-PKAc should conserve the anti-inflammatory role characteristic of PKA. Surprisingly, we observed the opposite. DnaJ-PKAc promotes inflammation by promoting infiltration of neutrophils and macrophage polarization to a pro-inflammatory microenvironment. Also, the ability to visualize the interaction of different immune cells with hepatocytes keeps surprising me. It is fascinating to realize how dynamic this microenvironment is during early stages of liver disease, a scenario that is remarkably similar to what we are used to observing during wound responses in the tail for example.

**Describe what you think is the most significant challenge impacting your research at this time and how will this be addressed over the next 10 years?**

The therapeutic options in the liver cancer field are very limited and not that efficient. In the case of FLC, it is particularly challenging that the oncogene DnaJ-PKAc carries scaffolding and kinase activity functions. This means that in order to develop efficient therapeutic approaches for FLC patients we probably need to develop combined treatments and target the dual function of DnaJ-PKAc. A zebrafish model like ours can help to accelerate the rate of drug discovery by testing thousands of compounds and different combinations in a small amount of time. Another challenging feature of FLC is its variability among patients. There are patients that display a ‘pure’ FLC phenotype and others with ‘mixed’ features of, for example, cholangiocarcinoma, which is very puzzling in the field. To better interpret these macroscopic differences, personalized medicine approaches could be one of the steps to try to correlate such differences with patients’ tumor aggressiveness, inflammation, immunogenicity and response to different treatments. Zebrafish avatars will undoubtedly be something to explore in the near future and provide clinical teams with additional information that might be extremely relevant at the time to choose patients treatment.

**Figure:**
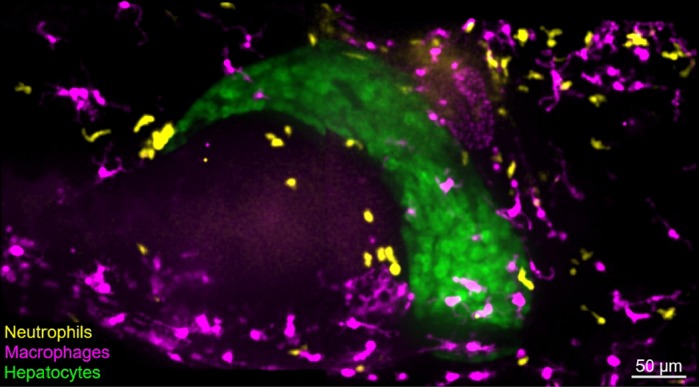
**Non-invasive live imaging of the liver microenvironment during early stages of fibrolamellar carcinoma development in a zebrafish larvae model.**

**What changes do you think could improve the professional lives of early-career scientists?**

For me to be ‘raised’ in a healthy and supportive environment during my PhD, and later during my postdoc, was crucial to achieve success and find my place in science and stay in science! Early-career scientists need to be helped to find a balance between their personal life and their career, and it is extremely hard to do that. When I finished my PhD (and my postdoc) I was completely burned out. I think this is where our scientific community really needs to gather and simply do more, from an institutional to a mentor standpoint. Yes, I know that I have achieved my goals but at what cost will be my question for the remaining years. There are universities and institutions that are taking seriously the mental health of their young scientists and developing offices with the goal of helping them to go through the most challenging periods of their lives. This is a subject that needs to be addressed ASAP in our community.

**What's next for you?**

I am currently in a very exciting phase of my career. I just started my new position as an assistant professor at Albert Einstein College of Medicine in the Department of Developmental and Molecular Biology. I am also one of the members of the Marion Bessin Liver Research Center, one of the oldest liver research centers in the USA, supported by the National Institute of Diabetes and Digestive and Kidney Diseases (NIDDK). Being surrounded by notable researchers such as Richard Stanley, Ana Maria Cuervo, Margaret Kielian or Robert Singer is extremely motivating for a junior scientist, and to be mentored by them is for sure a once-in-a-lifetime opportunity. At Einstein, I am developing an independent research program focused on understanding how diet impacts the immune system and how such effect is translated to liver disease progression. I am also extremely excited about the opportunity to use zebrafish models to study how diet-induced trained immunity affects inflammatory response towards a tissue damage or an infection. I believe that the effect of diet on reprogramming our immune system has drastic repercussions in the modulation of different human diseases, and zebrafish models will be extremely powerful to address such impact in a whole-animal context.

“[…] young female scientists, remember that you are not the only one, and it is achievable!”

**Any advice for young scientist that want to proceed a career in science?**

My advice is mostly for young female scientists… I never imagined myself doing anything other than being a scientist and, more importantly, being a female leader scientist! I think it was all about the environment I was ‘raised’ in – in Lisbon, at the Instituto of Molecular Medicine – with so many successful female leaders around me. It was just natural, so to become one of them sounded achievable – more than that, normal! I confess the doubts only appeared in my head when I came to the USA and mostly after I gave birth to my son 3.5 years ago. It was indisputably the most difficult and challenging period of my life. Suddenly I started to notice the lack of female scientist leaders everywhere – meetings, seminars, workshops, faculty positions (in almost every single institution). I am a very positive and determined person by nature but suddenly this made me realize how at a disadvantage I was day after day by not sleeping at night, not being able to stay in the lab as many hours as I wanted, and the constant fear of not publishing as much I was supposed to. Those were devastating moments. I could not see it at the time, but it was all about having the right support, family, PI, friends, etc, to overcome that period. I started to search for opportunities to promote my work, my ideas and goals. Your PI will be crucial during this process, so choose widely and ask people directly about this type of support when you're interviewing for PhD or postdoc positions. Also, work hard on building an efficient network from day 1! Don't be afraid to introduce yourself to junior and senior scientists; most of them enjoy a nice chat with younger scientists full of energy and ideas. It will make all the difference by the time you're applying for jobs. Until we find better ways to support young female scientists, remember that you are not the only one, and it is achievable!
